# Optimizing tACS for working memory: differential outcomes in healthy aging and non-amnestic mild cognitive impairment

**DOI:** 10.1186/s13195-025-01922-4

**Published:** 2025-12-02

**Authors:** Kristína Mitterová, Monika Pupíková, Martin Gajdoš, Ilona Eliášová, Irena Rektorová

**Affiliations:** 1 Applied Neuroscience Research Group, Brain and Mind Research Programme, Central European Institute of Technology, Masaryk University, Pekarska 53, Brno, 656 91 Czechia; 2 Multimodal and Functional Imaging Laboratory, Brain and Mind Research Programme, Central European Institute of Technology, Masaryk University, Brno, Czechia; 3 First Department of Neurology, St. Anne’s University Hospital and Faculty of Medicine, Masaryk University, Brno, Czechia

**Keywords:** TACS, Non-invasive brain stimulation, *n*-back task, Theta stimulation, Working memory, Healthy elderly, MCI

## Abstract

Transcranial alternating current stimulation (tACS) in the theta frequency range has been shown to enhance working memory (WM) performance. However, no studies have directly compared the effects of theta tACS between cognitively healthy elderly (HE) subjects and subjects with non-amnestic mild cognitive impairment (MCI). Our proof–of-concept study investigated the effects of monofocal (frontal) and bifocal (frontoparietal) theta tACS on WM in two cognitive loads in HE subjects and in subjects with MCI. In this sham-controlled, single-blinded, repeated-measures study with counterbalanced stimulation order across subjects (*n* = 55), theta tACS (4.51 Hz, 1.5 mA, 20 min) was applied either over the frontal site alone or simultaneously over frontal and parietal sites. WM 2-back and 3-back tasks were performed during and after each stimulation session. In the HE group, both frontal and frontoparietal stimulations improved performance, albeit with load-dependent differences. Frontal stimulation was particularly effective in the higher cognitive load, enhancing accuracy (β = -3.87; *p* = .033) and reaction times (β = − .042; *p* = .002) in the 3-back task. Frontoparietal stimulation improved accuracy (β = -3.74; *p* = .027) but not reaction time (*p* > .22) in the 2-back task in the HE group. Frontoparietal stimulation enhanced accuracy in the 3-back task across all participants (β = 1.91; *p* = .043). In the MCI group, frontal stimulation led to faster reaction times in the 3-back task, although the effects were not robust. Lastly, a marginally significant improvement in reaction times was observed in a letter 2-back transfer task following frontal stimulation (β = − .034; *p* < .092) across all participants. Our findings indicate that theta tACS over the frontal and frontoparietal areas elicits benefits in WM performance, driven mainly by enhancements in HE subjects. The effects of stimulation varied with cognitive load and montage, suggesting that optimal stimulation parameters may differ depending on task demands. The non-amnestic MCI group did not exhibit greater improvements despite their lower baseline performance, possibly due to higher variability in pathology and compensation. Multiple sessions or alternative stimulation parameters may be needed to achieve robust effects in subjects with MCI. The study was retrospectively registered on ClinicalTrials.gov (NCT06563453).

**Supplementary Information:**

The online version contains supplementary material available at 10.1186/s13195-025-01922-4.

## Background

Working memory (WM) is a multifaceted cognitive process that involves actively maintaining and manipulating information. It relies on a network of synchronized oscillatory activity across neuroanatomical hubs, including the prefrontal cortex and the posterior parietal, temporal, and occipital regions. The prefrontal cortex executes top-down control and facilitates the short-term retention and manipulation of information [1, 2]. The superior and inferior parietal lobules are involved in the temporal manipulation of information [3, 4], collaborating with the occipital cortex, which encodes visual memory elements [2, 5]. The medial temporal cortex contributes to WM by supporting binding associative processes and the integration of working and long-term memory systems [6–8]. Coordination of brain activity across these regions, crucial for optimized executive function performance, is regulated by the centralized hub of the frontoparietal control network (FPCN) [9], particularly through slower-band oscillations in the theta-alpha range (4–14 Hz), which are essential for long-distance neuronal synchronization [10, 11]. In addition, theta oscillations help organize local neural activity by modulating gamma bursts, with neurons firing in the gamma range nested at the troughs of the theta wave [11, 12]. Frontoparietal theta synchrony has been observed during cognitively demanding WM tasks [2] and is also implicated in specific WM components, including information maintenance, manipulation [13, 14], and retention [11].

Transcranial alternating current stimulation (tACS), a form of well-tolerated noninvasive brain stimulation, allows for the targeted modulation of multiple brain regions using oscillatory electric currents, increasing our understanding of the distributed nature of WM. By creating a spatially confined electric field [15, 16], tACS has the potential to entrain frequency-specific oscillations in targeted brain regions. By enhancing synchronization between sites, tACS is thought to promote optimal cross-regional coupling, improving network communication and efficiency [17–19]. Recent studies aiming to enhance WM have increasingly targeted theta oscillations in particular, using various tACS protocols. A promising approach involves bifocal stimulation over prefrontal and parietal regions to entrain frontoparietal theta activity. This method improved WM in healthy individuals, highlighting the causal role of synchronized theta connectivity within the FPCN. Notably, performance gains were observed specifically with in-phase (0° lag) compared to anti-phase (180° lag) stimulation [13, 18]. In a recent study, Pupíková et al. [19] compared monofocal and bifocal theta-tACS montages with theta-gamma cross-frequency coupling over the FPCN in healthy older individuals, and observed that different montages enhanced distinct aspects of *n*-back task performance. In more detail, monofocal frontal theta stimulation had a more positive effect on accuracy than sham stimulation did; bifocal frontoparietal in-phase theta stimulation led to faster reaction times, but did not affect accuracy. Theta-gamma phase-amplitude coupling also improved response times; however, there was some speed-accuracy trade-off depending on the protocol. Non-interventional EEG studies support this, showing that theta power increases with memory load [20] and is positively correlated with WM performance [21]. Given that both monofocal and bifocal theta stimulation protocols have been shown to enhance WM and increase theta-band spectral power [22] in healthy individuals, such approaches may also be effective in individuals with non-amnestic MCI. Targeting this early compensatory phase with neuromodulatory intervention could be critical for preserving cognitive function and potentially slowing the progression of cognitive decline.

Some studies have already demonstrated the effects of theta tACS on cognition in neurological patients, such as those with Parkinson’s disease [23]; other studies have argued that patient groups may benefit less than healthy subjects from tACS [24] or other non-invasive brain stimulation modalities [25]. Within the patient population, subjects with MCI tend to exhibit more consistent cognitive improvements following stimulation than subjects with advanced stages of cognitive impairment. To resolve these inconsistent results, the current study assessed the efficacy of monofocal and bifocal tACS protocols in a placebo-controlled, within-subject design, involving both healthy older subjects and subjects with MCI.

Studies in amnestic MCI subjects due to Alzheimer’s disease pathology have focused specifically on the benefits of gamma tACS applied over the precuneus [24], reporting improvements in associative memory [26, 27]. However, no study to date has examined the effectiveness of tACS in WM-relevant theta frequency in non-amnestic MCI patients. Taken together, this is the first study to examine whether monofocal or bifocal frontoparietal (in-phase) theta tACS—previously shown to enhance WM in cognitively healthy older adults—may hold therapeutic potential for individuals with MCI characterized by executive dysfunction [13, 19]. This proof-of-concept study examined both, the online changes in WM performance, and the immediate offline aftereffects of the stimulation to assess sustainability and transferability of the induced behavioral changes. Hypothesis: Stimulating a network associated with WM (FPCN) with a monofocal theta frequency leads to greater behavioral (offline and online) effects than bifocal stimulation. We expect the effects of theta-tACS to be greater in healthy elderly (HE) than in MCI individuals, as HE maintain more intact neural networks and oscillatory dynamics, allowing for more efficient entrainment and synchronization. Although MCI is associated with disrupted long-range connectivity during executive and WM tasks [28], preserved neuroplasticity suggests they may still benefit from targeted neuromodulation—making this an important and understudied group for intervention.

## Methods

### Study design and procedure

**Fig. 1:**
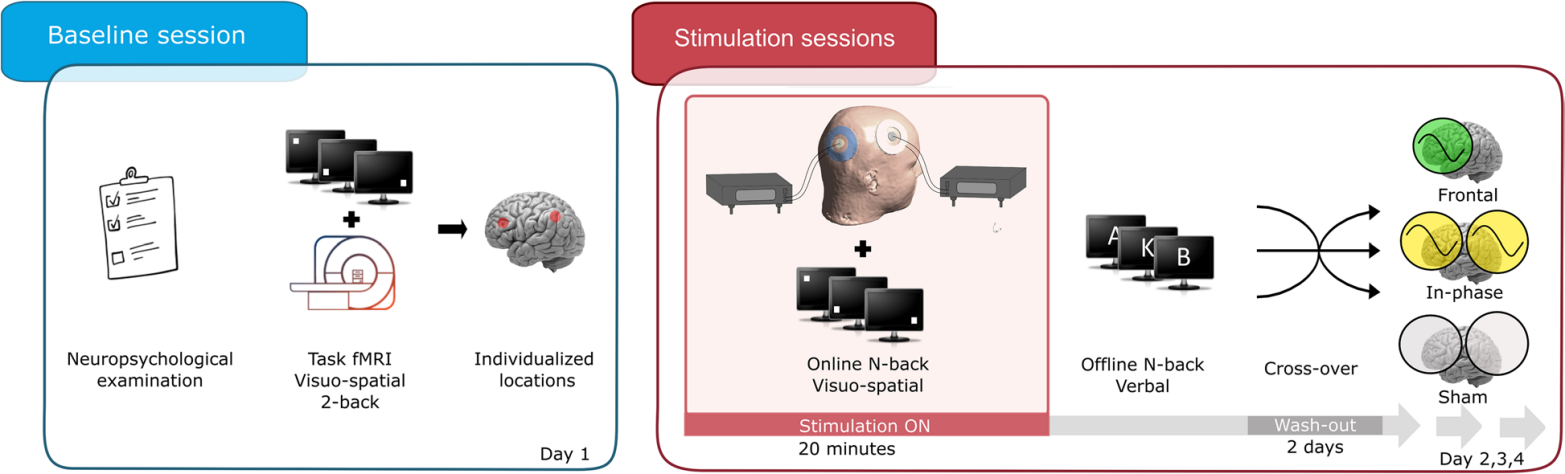
Design of the study

This randomized, within-subject, placebo-controlled study builds on our previous experiment described in Pupíková et al. [19] assessing the effects on WM-task performance of FPCN network neuromodulation by means of mono- or bifocal theta tACS. The primary outcome was the performance (task accuracy and response times during correct trials) during a visuospatial *n*-back task conducted online during the stimulation. The secondary outcome was the offline letter *n*-back task performance evaluated immediately after the stimulation. As depicted in Fig. 1, participants received monofocal stimulation of the frontal areas and synchronized bifocal frontoparietal stimulation and sham stimulation in three separate sessions. In each of the three protocols, we stimulated subjects with 1.5 mA at a lower theta frequency over the middle frontal gyrus (MFG) and, with the frontoparietal stimulation, also over the inferior parietal lobule (IPL). Based on the results of our previous study [19], we utilized the frontoparietal montage with an in-phase design; the MFG and IPL were synchronized with a relative phase of 0⁰, which has been shown to better promote cognitive function than an antiphase synchronization [17–19, 29–31]. The selection of the MFG and IPL regions was based on non-invasive brain stimulation studies which successfully improved WM (2-back task) when targeting these representative FPCN regions [13, 19] and a metaanalysis showing activations in these regions during n-back across three age groups [32].

Sessions were randomized and counterbalanced in their order for all participants and HE/MCI groups and separated by at least 48 h. Prior to the stimulation sessions, participants underwent baseline task-fMRI measurements to personalize the stimulation in terms of target location. The study was retrospectively registered on August 19, 2024, on ClinicalTrial.gov under NCT06563453.

### Participants

Altogether, the study sample consisted of 55 subjects (17 subjects with MCI with executive and visuoperceptual deficits and 38 HE subjects), none of whom had participated in our previous study by Pupíková et al. [19]. The subjects were examined upon recruitment using the neuropsychological battery detailed in Supplementary Table S1. Inclusion criteria for the HE group included normal cognitive performance as assessed by cognitive evaluation. Inclusion criteria for both groups were right-handed individuals with age over 60 years. A complex neuropsychological cognitive battery was used to evaluate five cognitive domains: memory, attention, executive functions, visuospatial functions, and language. Participants were also screened for signs of depression using the clinician’s interview and the Geriatric Depression Scale (GDS); only those without a major depressive episode were included in the study. Exclusion criteria for all participants: presence of dementia as assessed by a cognitive test battery and the assessment of activities of daily living, any major psychiatric disorder, history of another neurological disease affecting the central nervous system (e.g., tumor, epilepsy, stroke, etc.), severe or repeated head injury, non-compensated internal or oncological disease, and MRI-incompatible metal in the body (e.g., cardio stimulator). Participants were classified as MCI based on the presence of executive or visuospatial deficits (at least two tests below −1 SD) as compared with the age-appropriate norm; HE subjects had no objective cognitive deficit. In the MCI subjects, core clinical symptoms of dementia with Lewy bodies (DLB) and plasma biomarkers of Alzheimer’s disease pathology (NfL and pTau 181, 217, 231) were also examined [33–36], see Supplementary materials.

This study was approved by the Research Ethics Committee at Masaryk University (approval number EKV-2020–072). All participants provided written informed consent before data acquisition. The study was conducted in accordance with the Declaration of Helsinki and relevant ethical guidelines.

### Task design and procedure

**Fig. 2:**
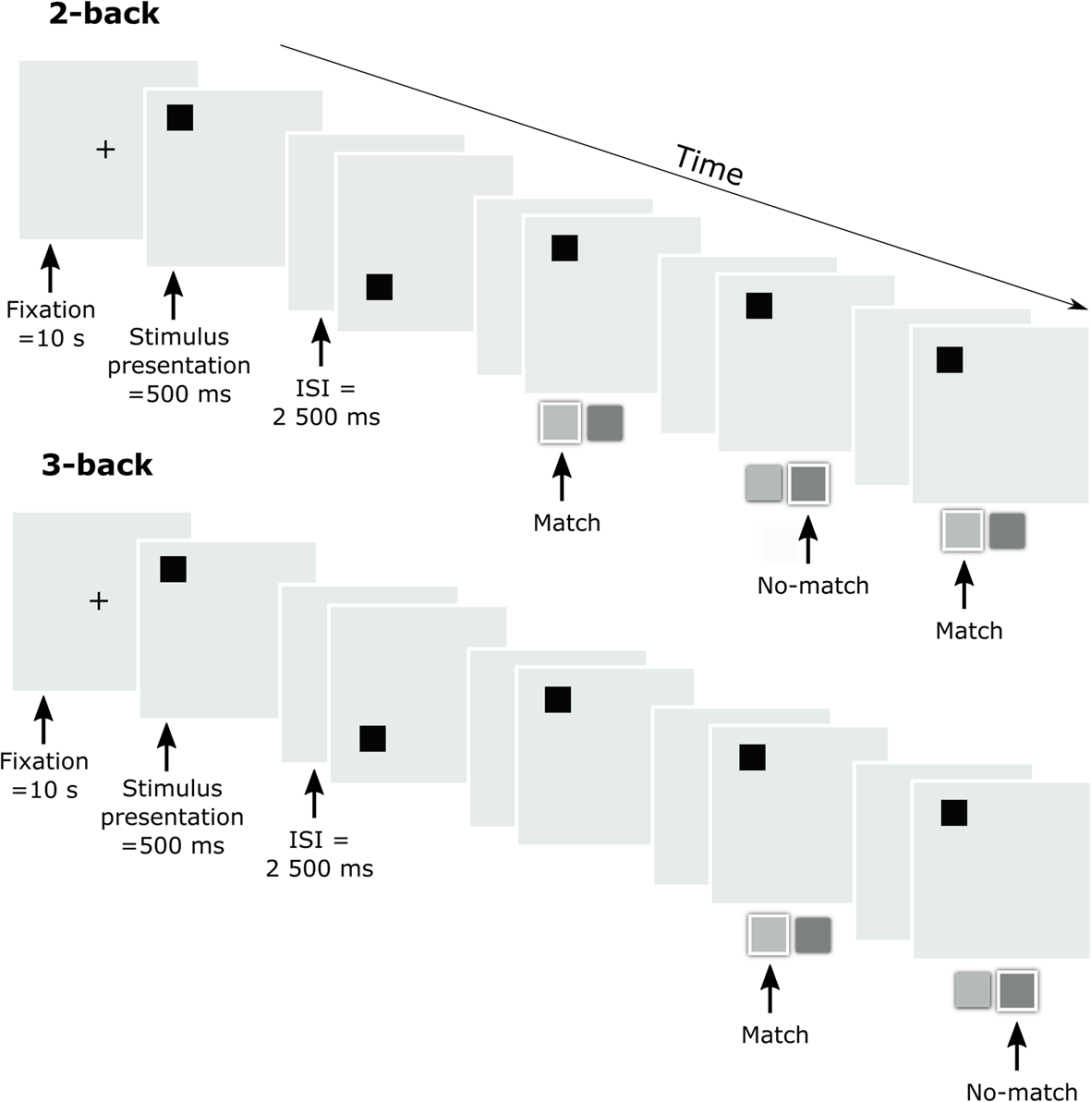
Visuospatial n-back task; task performed online during each stimulation condition. Participants completed two levels of difficulty during the stimulation. Participants indicated whether the current stimulus matches the one from *n* steps (2-back/3-back) earlier in the sequence. The task was also performed during fMRI to spatially individualize stimulation targets based on each participant’s activation

The main behavioral outcome measure, a visuospatial *n*-back task, was modified from Kramer et al. [37] with two levels of difficulty [19]. Participants viewed a stream of squares and were asked to indicate whether the square position matched the one from *n* steps (2-back/3-back) earlier in the sequence. All participants were administered two difficulty levels sequentially, with each participant starting with the easier 2-back task and continuing with the more difficult 3-back task. Black squares appeared in any of nine different positions. The stimulus was present on the screen for 500 ms with an inter-stimulus interval of 2500 ms. Participants responded using a response pad, pressing “YES” (left button) if the current stimulus matched the one presented *n* items earlier or “NO” (right button) if it did not. The online behavioral version of the task consisted of 100 trials divided into 10 blocks per difficulty level. At the beginning of each block, a fixation cross was displayed for 10 s (see Fig. 2). The baseline fMRI version consisted of 187 trials divided into 17 blocks of the 2-back difficulty only. Both difficulty levels were practiced by the participants during the baseline (opening) session to prevent high learning effects between the first and second stimulation sessions.

Within 15 min after the stimulation session, participants performed an offline verbal *n*-back task, adapted from Braver et al. [38], as a transfer task. This task followed the same procedure as the visuospatial task but used letters as stimuli. Participants completed one block of each difficulty level (2-back/3-back) in ascending order of difficulty, with each block comprising 10 trials. For all participants, accuracy and reaction times (for correct responses) were recorded for both difficulty levels across all blocks.

### Transcranial alternating current stimulation (tACS)

tACS was administered through two battery-driven stimulators attached to concentric electrodes placed over the task-fMRI individualized areas (see Supplementary materials) of the right middle frontal gyrus (rMFG) and right inferior parietal lobule (rIPL) for 20 min. A continuous, sinusoidal 4.51 Hz stimulation was applied at an intensity of 1.5 mA peak-to-baseline. This specific theta frequency was selected as the mean frequency based on our study in cognitively healthy subjects, who exhibited very similar low theta frequency with minimal variability ranges during concurrent EEG *n*-back task performance [19]. Protocols included monofocal theta stimulation over the frontal site only and bifocal theta stimulation of both sites with a relative phase shift of 0° (in-phase). Each concentric electrode pair was connected to a separate stimulator (current source, see Fig. 1). A pair of concentric electrodes consisted of a smaller inner electrode, which is a target electrode (inner electrode Ø 20 mm) and a larger reference electrode around the target electrode (outer electrode: Ø 75 mm with a hole Ø 40 mm). The current flowed between the inner and outer electrode. For the monofocal stimulation protocol, electrodes were placed over both stimulation sites, despite only one of the stimulators being active. Electrode placement was guided by a two-step procedure. First, we determined individualized stimulation targets based on individual activation peaks during the baseline fMRI 2-back task, located in close proximity to the right MFG and IPL regions, ensuring that stimulation targeted functionally-relevant regions (see further sections for details). These task-specific activation peaks were co-registered with each participant’s T1-weighted anatomical scan using Brainsight frameless stereotactic neuronavigation to guide the exact location of the inner (target) electrode center in each individual. The waveform of the stimulation was sinusoidal without DC offset. The sham stimulation was applied with the same settings, but the stimulator was turned off after 30 s. For details see Supplementary materials. The impedance was kept below 20 kΩ.

### Electric field simulation

**Fig. 3:**
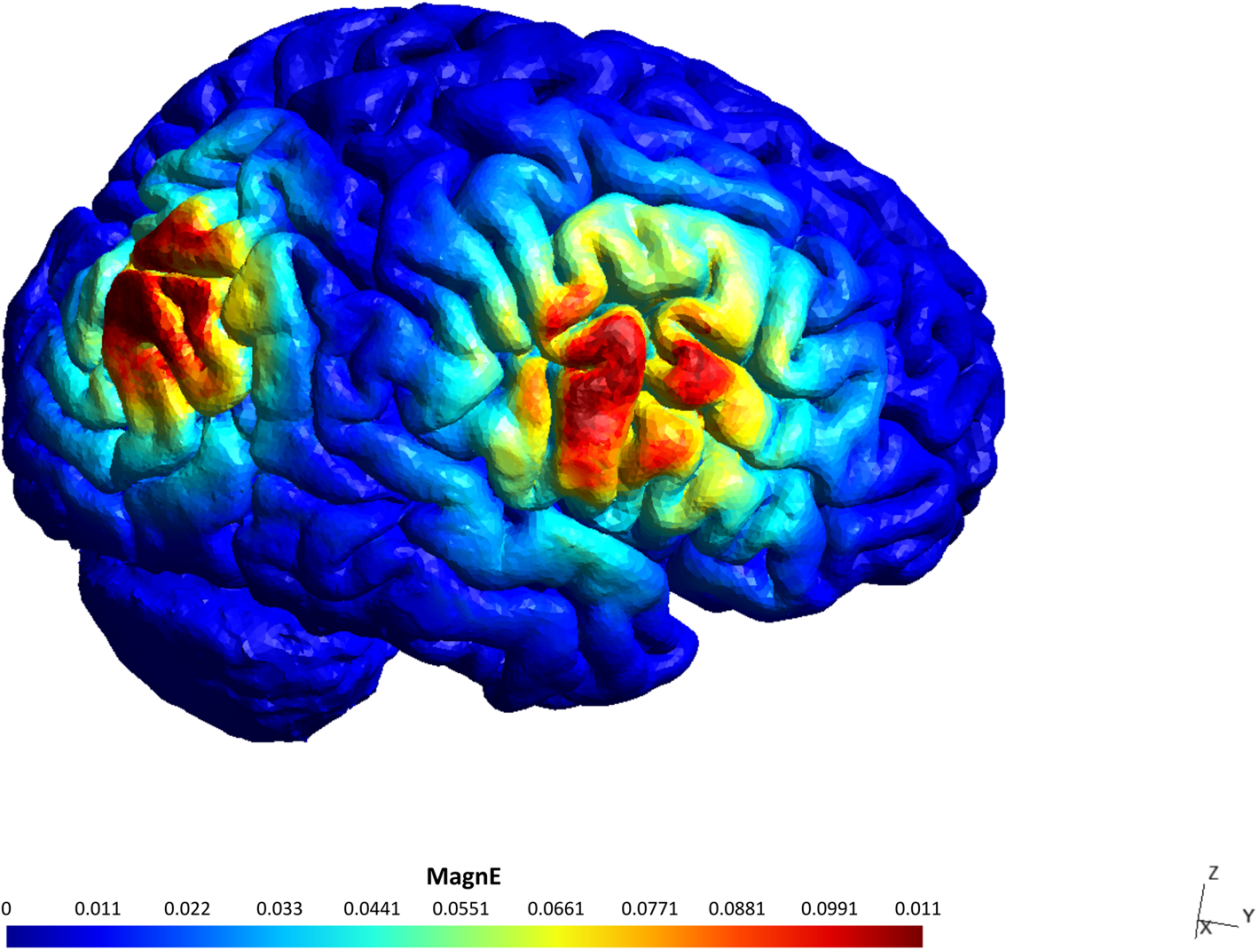
Example of an individual current density model determined using SimNIBS modeling software

We simulated the electric fields (EF) induced at each stimulation site for all participants a posteriori to estimate the influence of EF magnitude on task performance. Individual head models were constructed using SimNIBS [39], based on each participant's T1-weighted and, where available, T2-weighted MRI scans (Fig. 1). For each target site, we extracted the EF magnitude within a 10 mm radius, expressed as the mean value across the entire stimulated region of interest (ROI) (Fig. 3).

### MRI data acquisition and pre-processing

The MRI data was acquired via 3.0 T Magnetom Siemens Prisma. For the sMRI data, the T1 MPRAGE sequence (TR 2300 ms; TE 2.96 ms; voxel size 1 × 1 × 1 mm; FoV 256 × 256 mm; flip angle 9°; 192 transversal slices). Task-fMRI sequence info (task-fMRI; TR 1250 ms; TE 32 ms; voxel size 2 × 2 × 2 mm; FoV 224 mm; flip angle 65°; 76 transversal slices; 725 scans; multiband factor 4). The fMRI data was preprocessed and analyzed with SPM12 in MATLAB R2019a. The data preprocessing pipeline consisted of realign and unwarp, spatial normalization, and spatial smoothing (FWHM 6 mm). We controlled data for spatial abnormalities (e.g., dropouts) with the Mask Explorer tool [40] as well as for the artifacts related to the excessive head movement using framewise displacement (FD) criterion [41]. Each scan with FD > 1.5 mm was excluded in subsequent GLM analysis.

### Task fMRI analysis

We analyzed the task fMRI data with a general linear model (GLM) implemented in SPM12. The design matrix in the subject level analysis contained time courses of the task stimulation (a block design with an active condition and a baseline condition) convolved with a canonical hemodynamic function and head movement parameters (translations and rotations and their squares), with scans exceeding FD 1.5 mm marked as nuisance regressors (separate regressor for each suprathreshold scan).

The pre-stimulation task was used to identify the subject-specific coordinates for subsequent stimulation. We selected stimulation coordinates as the nearest local maxima to the right MFG (MNI: 36 28 36) and right IPL (MNI: 38 −50 36) coordinates on the parametric map of the active vs. baseline contrast, using a (voxel-level) threshold of *p* < 0.001. For variability in electrode placement see Supplementary Table S2.

### Statistical analyses

Between-group differences were evaluated for baseline descriptive statistics using t-tests and a chi-square test where appropriate. Next, we analyzed performance metrics (accuracy and reaction times during correct trials) separately for the 2-back and 3-back tasks using linear mixed models (LMMs) as a function of the stimulation condition (sham vs. frontal, sham vs. frontoparietal) and group (HE vs. MCI) to evaluate their interaction effect, while accounting for random effects of participants and sessions. For all performance outcomes, two LMMs were compared for fit using a likelihood ratio test (based on the chi-square distribution) in R:

$$\begin{aligned} \text{Baseline <- lmer}(\text{outcome}\;\sim\;&\text{Stim}\_\text{condition}\ast \text{group} \\& +(1\vert \text{Id}\_\text{subject})) \end{aligned}$$

$$\begin{aligned} \text{Full} <- &\text{lmer}(\text{outcome Stim}\_\text{condition}\ast \text{group}\\&+(1\vert \text{Id}\_\text{subject})+(1\vert \text{session}\_\text{nr})) \end{aligned}$$

To further explore the interaction effect, we conducted post-hoc analyses using the *emmeans* package in R. The offline *n*-back task changes were calculated in the same way. Cohen’s *d* was calculated across all blocks from the main effects when interactions were nonsignificant. The effects of age, gender, and education were assessed in separate models reported in Supplementary Table S9.

## Results

The HE (*n* = 38) and MCI groups (*n* = 17) differed in MoCA scores and education as well as in neuropsychological evaluation (Supplementary Table S1) but showed minimal differences in 2-back and 3-back performance markers (used as outcomes of interest; see Table 1) during the sham condition. MCI subjects exhibited elevated AD-related pathology, as indicated by plasma biomarker levels. They showed higher absolute concentrations of pTau231 and a significantly greater proportion of individuals exceeding the pathological cut-off for the pTau217 epitope (Supplementary Table S3). Altogether 9 (53%) of our MCI subjects met the criteria for possible or probable MCI-LB [42], i.e. they had one or two core features of DLB (Supplementary Table S4).

**Table 1:** Descriptive statistics of the sample

	Group	Mean	SD	t/χ^2^	*p*
MoCA	HE	27.39	1.67	3.77	<.001
	MCI	24.69	2.60		
Age	HE	68.37	5.76	.13	.899
	MCI	68.17	4.87		
Gender	HE	19/19		2.02	.155
	MCI	12/5			
Education	HE	15.83	3.51	2.53	.014
	MCI	13.47	2.29		
2-back ACC	HE	81.00	17.12	1.58	.119
	MCI	74.59	18.81		
2-back RT	HE	1.11	0.30	−0.35	.727
	MCI	1.14	0.33		
3-back ACC	HE	73.42	17.98	1.73	.089
	MCI	66.47	18.02		
3-back RT	HE	1.19	0.30	0.25	.802
	MCI	1.17	0.33		
	Group	Median	SD	W	***p***
Frontal mCD	HE	.09	.05	281	.542
	MCI	.10	.08		
Parietal mCD	HE	.05	.04	275	.676
	MCI	.05	.03		
Frontal pCD	HE	.17	.10	275	.471
	MCI	.18	.16		
Parietal pCD	HE	.11	.09	234	.660
	MCI	.12	.09		

2-back and 3-back performance was calculated from sham sessions; age and education in years; gender: female/male

*MoCA* Montreal Cognitive Assessment, *ACC* accuracy, *RT* reaction times during correct trials, *mCD* mean current density, *pCD* peak current density, *HE* cognitively healthy old subjects, *MCI* subjects with mild cognitive impairment, *SD* standard deviation, t = t-value; W = Mann–Whitney U test value; *p* = two-sided p-value

There were no significant differences in current density (V/m^2^) between the HE and MCI groups, as assessed using the Mann–Whitney U test (see Table 1). In an exploratory analysis, we also compared current density between tACS responders and non-responders in the frontal vs. sham and frontoparietal vs. sham conditions and observed no significant differences; current density was similar across groups and conditions (all *p* > 0.163).

### Online effects of theta tACS on n-back accuracy and reaction times

**Fig. 4:**
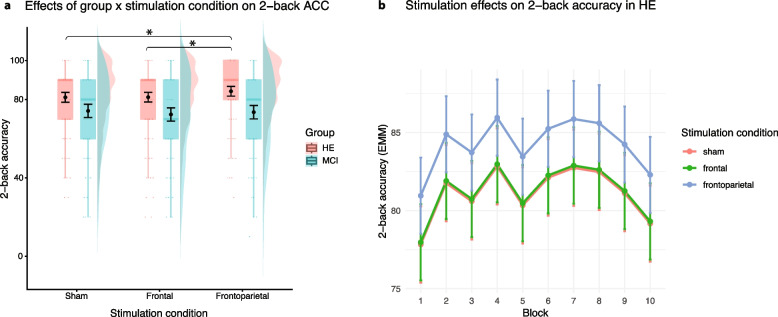
**a** Graph depicts distributions shown as violin plots, with boxplots indicating median and interquartile range. Black points with error bars represent estimated marginal means (EMM) with standard errors; * indicates significant contrasts after Tukey correction. **b** Graph depicts the main effect of stimulation condition on accuracy (ACC) in HE group. According to pairwise comparisons with Tukey correction, frontoparietal stimulation led to a small ACC mean increase of 3.3% (SE = 0.89, t(1085) = −3.76, *p* = 0.0005, d = 0.27) in comparison to sham. Plots were generated by ggplot2

LMM revealed a significant main effect of stimulation and a significant stimulation × group interaction for 2-back task accuracy. Participants performed significantly better during frontoparietal stimulation than during sham stimulation (*p* = 0.001). The effect of frontal stimulation was not significant (*p* = 0.954). The interaction effect suggests that the improvement during frontoparietal stimulation was specific to the HE group (Fig. 4; β = −3.74; SE = 1.69; *p* = 0.027; d = 0.84); performance in the MCI group did not differ significantly between the sham condition and any of the other stimulation conditions. Conditional *R*^2^ = 0.52 and marginal *R*^2^ = 0.06 suggest that random effects (i.e., subject variability and variability across sessions) capture a very large proportion of the variability in the data. Reaction times in the 2-back task did not improve in HE or MCI in comparison to sham (*p* > 0.22); see Supplementary Tables S5a and S5b for the 2-back task results.

**Fig. 5:**
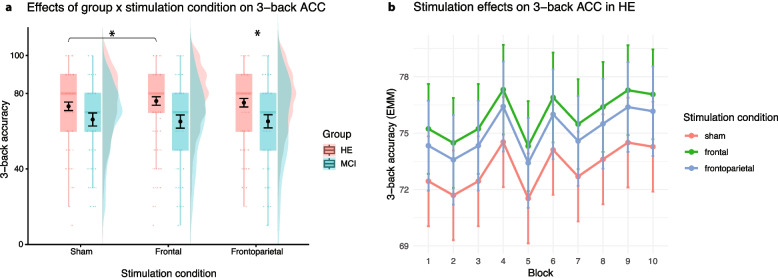
**a** Graph depicts distributions shown as violin plots, with boxplots indicating median and interquartile range. Black points with error bars represent estimated marginal means (EMM) with standard errors; * indicates significant contrasts after Tukey correction. **b** Graph depicts the main effect of stimulation condition on ACC in HE group. According to pairwise comparisons with Tukey correction, frontal stimulation led to mean increase of 2.8% in ACC (SE = 0.97, t(1589) = −2.82, *p* = 0.037, d = 0.21) in comparison to sham. Plots were generated by ggplot2

During the 3-back task, accuracy was significantly higher during the frontoparietal stimulation (β = 1.91; SE = 0.094; *p* = 0.043, d = 0.09) as compared to sham in all participants. The effect of the frontal stimulation was driven by significantly higher accuracy in the HE compared to the MCI group, as indicated by a significant interaction (β = −3.87; SE = 1.81; *p* = 0.033, d = 0.82). Similarly, conditional *R*^2^ = 0.49 and marginal *R*^2^ = 0.05 point to a very large proportion of the subject variability and variability across sessions in the data. In the MCI group, accuracy did not differ significantly between active stimulation conditions and the sham stimulation condition (Fig. 5).

**Fig. 6:**
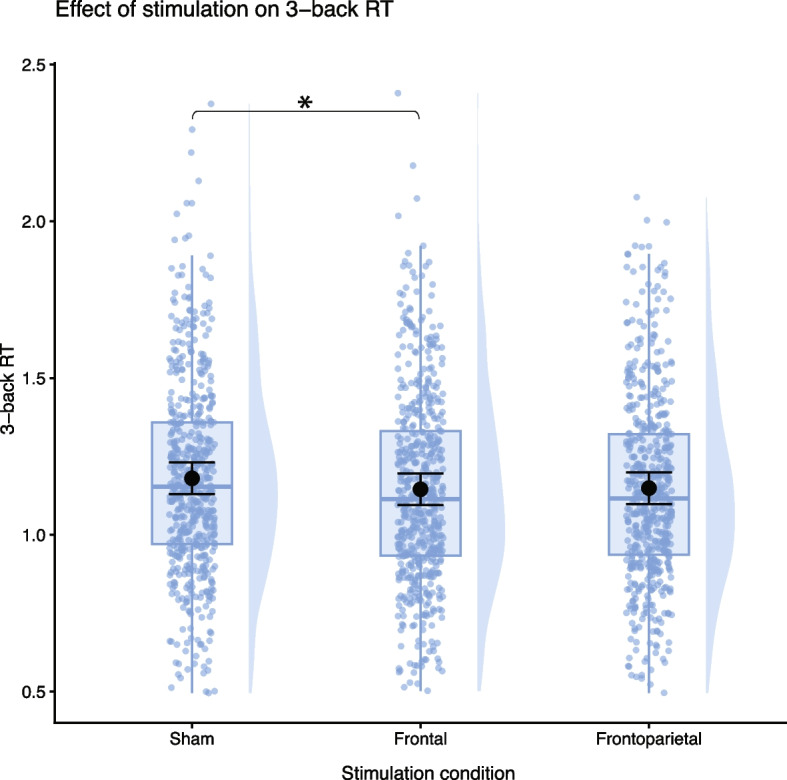
Graph depicts distributions shown as violin plots, with boxplots indicating median and interquartile range. Black points with error bars represent estimated marginal means (EMM) with standard errors of the main effect of stimulation conditions on block reaction times (RT), as the interaction between stimulation and group was not significant. According to pairwise comparisons with Tukey correction, frontal stimulation led to a mean decrease in RT of 0.034 s (SE = 0.01, t(1583) = 3.02, *p* = 0.007) compared to sham. Plots were generated by ggplot2

Similarly, reaction times during correct responses were significantly faster only during frontal stimulation across all participants as compared to sham (Fig. 6; β = − 0.042; SE = 0.014; *p* = 0.002, d = 0.18); subject variability and variability across sessions (conditional *R*^2^ = 0.62) explained significantly larger proportion of the variance in the data (marginal *R*^2^ = 0.05) compared to the fixed effects (see Supplementary Tables S6a and S6b for detailed 3-back results and Supplementary Table S7 for model comparisons).

### Offline effects of theta-phased tACS on *n*-back tasks

**Fig. 7:**
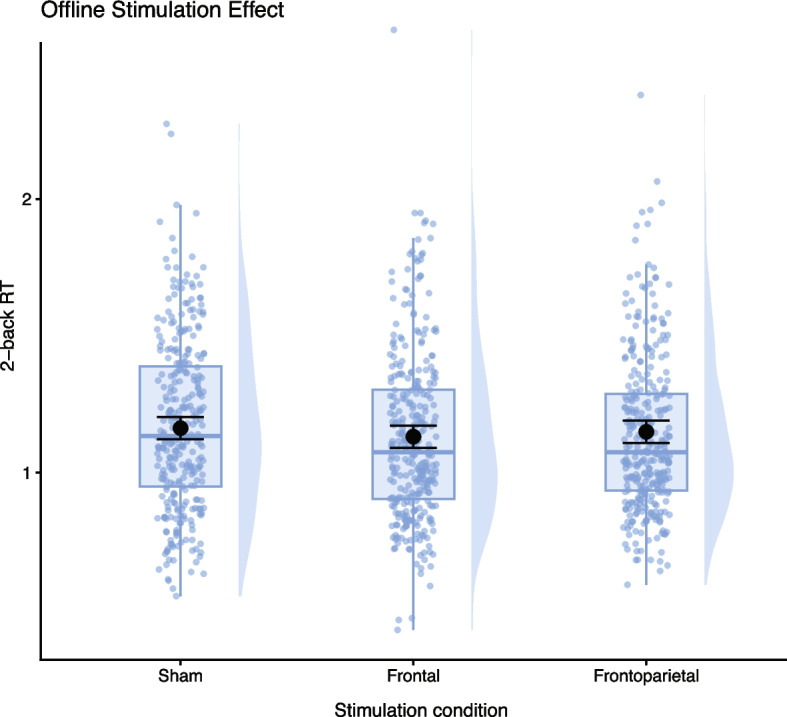
Graph depicts distributions shown as violin plots, with boxplots indicating median and interquartile range. Black points with error bars represent estimated marginal means (EMM) with standard errors of the main effect of stimulation conditions on block reaction times (RT), as the interaction between stimulation and group was not significant. According to pairwise comparisons with Tukey correction (a model without the interaction effect), frontal stimulation led to a mean decrease in RT of 0.03 s (β = −0.033, SE = 0.016, t(902) = 1.87, *p* = 0.148) compared to sham. Plots were generated by ggplot2

After the frontal stimulation, all participants demonstrated marginally significant faster reaction times on the offline letter 2-back task as compared to after sham stimulation (Fig. 7; β = − 0.034; SE = 0.020; *p* < 0.092, d = 0.14); all other offline performance metrics were non-significant (see Supplementary Table S8).

Each significant model contained influential cases identified by Cook’s distances; after removing these cases, all models were successfully refitted, resulting in the same or improved fit. Similarly, the results were unchanged when controlled for age, gender, and education, however the proportion of variance explained by the fixed factors increased (Supplementary Table S9).

## Discussion

Our results show that theta tACS over MFG (monofocal) and MFG and IPL (bifocal in-phase) can lead to significant improvements in WM task performance, depending on the cognitive load of the online task and the cognitive status of the subject.

The performance of HE subjects improved after both frontal and frontoparietal stimulations, albeit with load-dependent differences. The frontal montage was more effective for the higher cognitive load, with both accuracy and reaction times enhanced (shortened) in the 3-back task. The frontoparietal montage showed greater efficacy for the lower cognitive load than for the higher cognitive load, improving accuracy only for the 2-back task. Notably, reaction times in HE remained stable during the frontoparietal stimulation for the 2-back task, likely reflecting already optimized processing speeds and performance nearing a ceiling effect. This aligns well with previous studies in healthy older individuals, where frontoparietal synchronization by theta tACS improved WM performance when cognitive demands were moderately high (2-back level), but not overwhelming (3-back task) [13, 17]. In the MCI group, only frontal stimulation induced shorter reaction times during the 3-back task; however, these results were not robust, and the contrasts did not remain significant after Tukey correction. Therefore in this non-amnestic MCI group, alpha tACS of the occipital areas might prove more effective on executive functions, as was seen in a recently published study with a DLB patient cohort [43].

Regarding the observed effect in HE, our results align well with recent meta-analyses reporting small-to-medium-sized effects of tACS across all frequency bands [31] and specifically theta tACS [44], and further show that stimulation efficacy may also depend on the cognitive load of the online WM task used concurrently with tACS. The present study supported our previous findings that the frontal montage was more effective for the more difficult (3-back) task [19] compared to other frontoparietal theta and theta-gamma montages. Evidence suggests that older adults who achieve performance levels comparable to those of younger adults exhibit frontal compensatory over-activation, accompanied by weaker posterior activations [45], especially during short-term memory tasks [46, 47], and the recruitment of frontal regions increases with cognitive load [48]. In line with this notion, the frontal tACS may have successfully promoted functional compensation under particularly demanding cognitive conditions.

Despite the observed robust online effect, we observed only a marginally significant short-term after-effect of the frontal stimulation, manifested as faster reaction times in a transfer task (letter 2-back task). This may be attributed to the nature of the task, as the letter *n-*back also relies on the phonological loop, which is supported by left-lateralized language-processing regions such as the Broca area [49, 50]. Employing subvocal rehearsal during phonological processing or reorganization of information into a more familiar “chunks” can substantially increase WM [51]. This suggests that interindividual differences in using mnemonic strategies may have influenced performance on letter *n-*back tasks. Our stimulation targeted the right MFG and IPL and thus the modulation of neural processes specific to the phonological domain may have been limited. Another interpretation might be that one session of tACS induces only very mild aftereffects; this is partially confirmed in the literature [52]. We were not able to disentangle the lack of sustainability of the enhanced online effects from the lack of transfer of the behavior to another WM task.

We observed comparable current density across groups, with no significant differences between MCI and HE participants, nor between tACS responders and non-responders. This suggests that current density alone may not adequately capture individual variability in response to stimulation. A more valid mechanistic indicator of tACS-induced entrainment may be frequency-specific changes in neural activity. Supporting this, a recent study by Debnath et al. [22] demonstrated that theta-tACS improved WM reaction times and significantly increased post-tACS spectral power at 5 Hz, but not in other frequency bands. Future studies should incorporate neurophysiological markers, such as EEG-based spectral power or connectivity metrics, to directly assess the neural mechanisms underlying theta tACS effects and determine whether entrainment occurs even in the absence of overt behavioral improvement.

The modest magnitude of effects in the MCI subgroup could be attributed to the use of a single stimulation session and stimulation parameters derived from a healthy cohort. Specifically, the theta frequency of 4.51 Hz was derived from EEG recordings in our previous study on HE [19], it remains unclear whether this frequency is optimal for individuals with MCI or how much variability exists within this group. These factors may have limited the efficacy of tACS in individuals with greater neurobiological and cognitive heterogeneity. An alternative explanation for the positive effect observed in HE and the negative effect in non-amnestic MCI may lie in the choice of stimulation targets. Modulating multidomain FPCN hubs may enhance top-down cognitive control in HE, whereas in non-amnestic MCI it may be necessary to additionally target deep brain structures implicated in early disease pathology and involved in executive functions and working memory, such as the striatum [53–55]. Recent advances have made this feasible with novel stimulation approaches, including temporal interference electrical stimulation [56–59] and low-intensity focused ultrasound [60].

There are several limitations to the present study. First, the small sample size of MCI patients may have reduced the statistical power to detect the true effect of theta tACS in this group as compared to the more homogenous HE group. Heterogeneity in individual data was reflected in the discrepancy between marginal and conditional R^2^ values, where most of the variance was attributable by differences between subjects and sessions; this may be characteristic of the prodromal symptoms of DLB of our MCI cohort. Second, theta frequency and single session design were derived from our previous study on HE [19]. According to recent reviews [61, 62], individualized frequency protocols, such as stimulating at a theta frequency below the individual peak for enhancement of WM may be the most effective approach [63]. In line with this, one study found that event related synchronization during n-back task differed between MCI who cognitively declined within one year and healthy controls [64]. By contrast, another study reported such difference only under the most demanding (3-back task) condition [65].

This proof-of-concept single-session, randomized, placebo-controlled study suggests that theta tACS applied to the frontoparietal network hubs enhances cognitive function in HE. Multiple session studies are warranted to evaluate the durability and cumulative benefits of theta tACS. It remains unclear whether the absence of robust offline effects after one stimulation session reflects an insufficient transfer or the lack of a direct offline effect. Ensuring consistency in task design between online and offline assessments will help determine the generalization of tACS effects. Future work should also examine the impact of genetic variables such as APOE polymorphism or GBA mutation [66], and on the effect of individual resting-state fMRI/EEG connectivity [67] patterns on stimulation behavioral effects. Lastly, while gamma stimulation has shown promise for episodic memory in amnestic MCI, future studies comparing non-amnestic and amnestic subtypes could clarify frequency- and network-specific mechanisms of tACS.

To conclude, our proof-of-concept study demonstrates that theta tACS over the frontoparietal areas elicits benefits in WM performance in healthy older adults, with effects varying by cognitive load and stimulation montage. Frontal stimulation proved most effective under higher cognitive demands, improving both accuracy and reaction times; frontoparietal stimulation was more beneficial for lower-load tasks. In contrast, MCI participants showed limited and non-robust improvements. These findings may seem counterintuitive, as one might expect individuals with lower baseline performance to have greater potential for improvement with tACS treatment. Multiple explanations are possible. The MCI group exhibits greater variability in both underlying pathology and cognitive performance, as well as fluctuations in cognitive states. Additionally, compensatory mechanisms may modulate the effects of stimulation, making the impact of tACS less straightforward in MCI than in the relatively more homogeneous HE population. Multiple sessions or different stimulation parameters (such as stimulation frequency) may be necessary to elicit meaningful effects in this population and future studies should make this a key focus. These findings suggest that while theta tACS may help support cognitive performance in healthy aging, only repeated stimulation is likely to yield meaningful benefits for quality of life. Therefore, multi-session protocols are needed to validate the promising, but thus far short-lived, online effects observed in this population. Finally, closed-loop tACS systems that measure ongoing brain activity and apply real-time targeted stimulation may be the way forward for modulating brain function [68] particularly in cognitively impaired subjects.

## Supplementary Information

Supplementary Material 1

## Data Availability

Data are available upon request.

## Abbreviations

FPCN: Frontoparietal Control Network
HE: Healthy Elderly
IPL: Inferior Parietal Lobule
MCI: Mild Cognitive Impairment
MFG: Middle Frontal Gyrus
tACS: Transcranial Alternating Current Stimulation
WM: Working Memory
