# Supplementary material for: Optimizing tACS for working memory: differential outcomes in healthy aging and non-amnestic mild cognitive impairment
Source: Alzheimers Res Ther. 2025 Dec 2;18:2. doi: 10.1186/s13195-025-01922-4 (PMC12777262; doi:10.1186/s13195-025-01922-4)
Supplement: Supplementary file 1 — Supplementary Material 1 [file 13195_2025_1922_MOESM1_ESM.docx]

Supplementary materials

**Supplementary methods**

**1.1. Participant examination, the questionnaires and scales**

Participants were enrolled from a database of HE and prodromal dementia subjects and gave written consent to be potentially addressed by our researchers for future studies. A clinical and neurological examination involved detailed medical history, Unified Parkinson’s Disease Rating Scale: Motor Examination (UPDRS III) with a cut-off score > 3 points, Montreal Cognitive Assessment (MoCA) with a cut-off score < 26 or ≤ 26 depending on years of education, REM sleep behavior disorder screening questionnaire (RBDSQ) with a cut-off score ≥ 5 points, Mayo Fluctuation Scale (MFS) with a cut-off score ≥ 3 points, Neuropsychiatric Inventory (NPI) for detecting hallucinations, misperceptions, or psychosis (Y/N), Geriatric Depression Scale (GDS) with a cut-off score of 10 points, Epworth Sleepiness Scale (ESS) with a cut-off score of 11 points. Complex neuropsychological cognitive battery was used to evaluate five cognitive domains (see Supplementary Table S1). Standardized age normative Z-scores were calculated for each subject. Performance was considered as deficit if participants achieved scores in at least two tests below 1 SD from the age-appropriate norm.

**1.2. Transcranial alternating current stimulation protocols**

A single blinded design on the side of subjects was adopted. Manufacturer information: tACS was performed through a battery-driven stimulator (DS-5, Digitimer, Hertfordshire, United Kingdom Digitimer) attached to two pairs of concentric electrodes (NeuroConn GmbH, Germany). We used the T1 MRI scan-based frameless stereotactic neuro-navigation targeting with Brainsight 2 (Rogue Research Inc., Canada). The electrodes were held in place by conductive paste (Ten20 Conductive Paste gel, Weaver and Company, USA).

**Table S1** *Comparison of Cognitive Measures Between HE and MCI Groups*

| Test | M (SD) | | *p* |
| --- | --- | --- | --- |
|  | **HE** | **MCI** |  |
| Judgement of Line Orientation | 0.47 (.73) | -0.59 (1.22) | .001** |
| Brief Visuospatial Memory Test – short memory | 0.71 (.86) | -0.63 (1.17) | <.001** |
| Brief Visuospatial Memory Test – delayed memory | 0.67 (.63) | -0.30 (1.12) | <.001** |
| Philadelphia Verbal Learning Test – immediate memory | 0.46 (.94) | -0.54 (.98) | .003* |
| Philadelphia Verbal Learning Test – short memory | 0.38 (.84) | -0.34 (.77) | .013* |
| Philadelphia Verbal Learning Test – delayed memory | .34 (.97) | -.85 (1.02) | .001** |
| Digit Symbol Substitution Test | .42 (.78) | .10 (.80) | .190 |
| Digit Number Sequencing | .31 (.72) | -.06 (.79) | .101 |
| Lexical Fluency | .50 (.94) | .06 (.98) | .136 |
| Semantic Fluency | .26 (.84) | -.40 (1.18) | .030* |
| Picture Arrangement | -.10 (.71) | -1.03 (.81) | .001** |

*Note*: test scores are in Z-score; HE = healthy old; MCI = mild cognitive impairment. M = mean; SD = standard deviation; p-values are two-sided.

**Table S2** *Comparison of Mean distance (mm) of individual ROIs from the group centroid for the middle frontal gyrus (MFG) and inferior parietal lobule (IPL), reflecting interindividual variability in ROI localization.*

|  | | **M (SD)** | | ***p*** |
| --- | --- | --- | --- | --- |
|  |  | **HE** | **MCI** |  |
| Spatial deviation (mm) | MFG | 11.53 (7.62) | 14.48 (8.41) | .204 |
|  | IPL | 10.48 (5.23) | 10.25 (4.14) | .872 |
|  | ***p*** | .412 | .100 |  |

**1.3. Plazma Biomarkers**

All our participants underwent plasma sample collection through venipuncture. Plasma NfL and pTau epitopes were evaluated by our partner institution (Clinical Neurochemistry Laboratory, University of Gothenburg, Sweden) in a blinded manner, on the Simoa HD-1 (Quanterix, Billerica, MA, USA) as described elsewhere (1) with cut-off scores based on ROC analyses reported in the literature (see Supplementary Table S2 for the respective cutoffs).

**Supplementary results**

**Table S3** *Comparison of Plasma Biomarkers Between HE and MCI Groups*

| **Marker** | **Group** | **M±SD** | ***t*** | ***p*** | ***n* of positive subjects** | **Fishers exact test *p*** |
| --- | --- | --- | --- | --- | --- | --- |
| NfL | MCI = 11 | 20.30 ± 4.49 | .424 | .674 | 9 | .066 |
|  | HE = 20 | 19.39 ± 7.46 |  |  | 9 |  |
| pTau181 | MCI = 11 | 8.63 ± 2.73 | 2.068 | .051* | 1 | .591 |
|  | HE = 20 | 6.48 ± 2.84 |  |  | 1 |  |
| pTau217 | MCI = 11 | 1.72 ± 1.34 | 1.608 | .130 | 3 | .037* |
|  | HE = 20 | 1.01 ± .81 |  |  | 0 |  |
| pTau231 | MCI = 11 | 13.02 ± 3.27 | 2.340 | .027* | 1 | .591 |
|  | HE = 20 | 9.81 ± 4.27 |  |  | 1 |  |

Plasma biomarkers positivity counts are based on the following cutoffs with references: NfL 17.58 pg/ml (2), pTau181 12.2 pg/ml (3), pTau217 2.50 pg/ml (4), pTau231 17.652 pg/ml (5), p-values are two-sided

**Table S4** *Core clinical features of DLB in the MCI Group* (6)

| Test | *n* of positive subjects |
| --- | --- |
| Motor features of parkinsonism (UPDRS III; cut-off > 3) | 4 |
| Recurrent visual hallucinations (Neuropsychiatric Inventory; cut-off = 1) | 0 |
| REM sleep behavior disorder (REM Sleep Behavior Questionnaire; cut-off ≥ 5) | 5 |
| Fluctuating cognition and alertness (Mayo fluctuation scale; cut-off ≥ 3) | 2 |

**Table S5a** *Results of the Linear Mixed Model for Accuracy in the 2-Back Task*

| **Predictor** | **Estimate** | **SE** | **df** | ***t*** | ***p*** |
| --- | --- | --- | --- | --- | --- |
| Intercept | 81.13 | 2.40 | 27.10 | 33.83 | < .001** |
| Target: frontal | .05 | .94 | 1567.53 | .06 | .954 |
| Target: frontoparietal | 3.08 | 0.93 | 1564.52 | 3.32 | .001** |
| Group | -6.92 | 3.74 | 62.83 | -1.85 | .069 |
| Target: frontal × MCI | -1.88 | 1.67 | 1566.15 | -1.12 | .262 |
| Target: frontoparietal × MCI | -3.74 | 1.69 | 1566.79 | -2.21 | .027* |

*Note:* Target: sham is a reference category, Group has two levels: HE (reference in interactions) and MCI. SE = Standard Error, df = Degrees of Freedom, t = t-value, p = p-value.

**Table S5b** *Results of the Linear Mixed Model for Reaction Times in the 2-back Task*

| **Predictor** | **Estimate** | **SE** | **df** | ***t*** | ***p*** |
| --- | --- | --- | --- | --- | --- |
| Intercept | 1.102 | 0.052 | 14.17 | 21.20 | < .001** |
| Target: frontal | 0.007 | 0.014 | 1565.00 | 0.52 | .604 |
| Target: frontoparietal | 0.004 | 0.013 | 1564.00 | 0.30 | .767 |
| Group | 0.045 | 0.072 | 59.11 | 0.63 | .533 |
| Target: frontal × MCI | -0.019 | 0.024 | 1564.00 | -0.80 | .427 |
| Target: frontoparietal × MCI | -0.030 | 0.025 | 1565.00 | -1.21 | .228 |

*Note:* Target: sham is a reference category, Group has two levels: HE (reference in interactions) and MCI. SE = Standard Error, df = Degrees of Freedom, t = t-value, p = p-value.

**Table S6a** *Results of the Linear Mixed Model for Accuracy in the 3-back Task*

| **Predictor** | **Estimate** | **SE** | **df** | ***t*** | ***p*** |
| --- | --- | --- | --- | --- | --- |
| Intercept | 73.17 | 2.19 | 42.43 | 33.37 | < .001** |
| Target: frontal | 2.80 | 0.95 | 1585.24 | 2.96 | .003* |
| Target: frontoparietal | 1.91 | 0.94 | 1584.16 | 2.03 | .043* |
| Group | -7.01 | 3.88 | 63.80 | -1.81 | .076 |
| Target: frontal × MCI | -3.87 | 1.81 | 1585.40 | -2.14 | .033* |
| Target: frontoparietal × MCI | -2.89 | 1.84 | 1587.85 | -1.57 | .117 |

*Note:* Target: sham is a reference category, Group has two levels: HE (reference in interactions) and MCI. SE = Standard Error, df = Degrees of Freedom, t = t-value, p = p-value.

**Table S6b** *Results of the Linear Mixed Model for Reaction Times in the 3-back Task*

| **Predictor** | **Estimate** | **SE** | **df** | ***t*** | ***p*** |
| --- | --- | --- | --- | --- | --- |
| Intercept | 1.180 | 0.051 | 12.46 | 23.10 | < .001*** |
| Target: frontal | -0.042 | 0.014 | 1581.75 | -3.11 | .002** |
| Target: frontoparietal | -0.019 | 0.014 | 1581.16 | -1.44 | .151 |
| Group | -0.009 | 0.073 | 59.53 | 0.12 | .902 |
| Target: frontal × MCI | 0.026 | 0.026 | 1581.61 | 1.00 | .316 |
| Target: frontoparietal × MCI | -0.047 | 0.026 | 1582.99 | -1.78 | .075 |

*Note:* Target: sham is a reference category, Group has two levels: HE (reference in interactions) and MCI. SE = Standard Error, df = Degrees of Freedom, t = t-value, p = p-value.

**Table S7** *Model comparison*

| **Model** | **AIC** | ***χ2 (df)*** | ***p*** |
| --- | --- | --- | --- |
| 2-back ACC interaction + (1 \| Id_subject) | 13048 | 26.67(1) | < .001 |
| *2-back ACC interaction + 2 random effects | 13023 |  |  |
| 3-back ACC interaction + (1 \| Id_subject) | 13347 | 9.89(1) | .002 |
| *3-back ACC interaction + 2 random effects | 13339 |  |  |
| 3-back RT interaction + (1 \| Id_subject) | -437.26 | 119.3(1) | < .001 |
| *3-back RT interaction + 2 random effects | -554.55 |  |  |

*Note:* *models with significantly better fit, AIC = Akaike Information Criterion goodness of fit, X^2^(df) = Chi-square with degrees of freedom, p = p-value

**Table S8** *Results of the Linear Mixed Model for Reaction Times in the offline 2-back Task*

| **Predictor** | **Estimate** | **SE** | **df** | ***t*** | ***p*** |
| --- | --- | --- | --- | --- | --- |
| Intercept | 1.157 | 0.045 | 26.20 | 25.70 | < .001** |
| Target: frontal | -0.034 | 0.020 | 901.84 | -1.69 | .092 |
| Target: frontoparietal | -0.011 | 0.020 | 900.72 | -0.53 | .594 |
| Group | 0.017 | 0.070 | 65.53 | -0.25 | .805 |
| Target: frontal × MCI | 0.010 | 0.036 | 900.37 | 0.28 | .784 |
| Target: frontoparietal × MCI | -0.009 | 0.036 | 901.40 | -0.26 | .799 |

*Note:* Target: sham is a reference category, Group has two levels: HE (reference in interactions) and MCI. SE = Standard Error, df = Degrees of Freedom, t = t-value, p = p-value.

**Table S9** *Results of the Linear Mixed Model for both n-back tasks cleaned off the effect of age, education and gender.*

| **Predictor** | **Estimate** | **SE** | **df** | **t** | **p** |
| --- | --- | --- | --- | --- | --- |
| **2-Back Accuracy** |  |  |  |  |  |
| Intercept | -.98 | 2.67 | 35.89 | -.37 | .717 |
| Target: frontal | .05 | .94 | 1567.15 | .05 | .958 |
| Target: frontoparietal | 3.08 | .93 | 1563.87 | 3.33 | .001 ** |
| Group | -5.31 | 3.40 | 64.77 | -1.56 | .123 |
| Gender (M) | 3.76 | 3.04 | 54.85 | 1.24 | .221 |
| Target: frontal × MCI | -1.87 | 1.67 | 1565.47 | -1.12 | .264 |
| Target: frontoparietal × MCI | -3.73 | 1.69 | 1566.52 | -2.21 | .028 * |
| **3-back Accuracy** |  |  |  |  |  |
| Intercept | -1.83 | 2.53 | 52.23 | -.73 | .472 |
| Target: frontal | 2.70 | .97 | 1583.51 | 2.78 | .006 * |
| Target: frontoparietal | 2.27 | .97 | 1583.92 | 2.35 | .019 * |
| Group | -5.23 | 3.40 | 66.01 | -1.54 | .128 |
| Gender (M) | 5.46 | 3.02 | 54.94 | 1.81 | .076 |
| Target: frontal × MCI | -3.09 | 1.75 | 1583.11 | -1.77 | .078 |
| Target: frontoparietal × MCI | -3.79 | 1.77 | 1586.91 | -2.14 | .033 * |
| **2-back reaction times** |  |  |  |  |  |
| Intercept | .09 | .06 | 19.62 | 1.56 | .134 |
| Target: frontal | .01 | .01 | 1567.15 | .53 | .594 |
| Target: frontoparietal | .00 | .01 | 1563.87 | .30 | .766 |
| Group | .00 | .06 | 60.09 | .05 | .959 |
| Gender (M) | -.17 | .06 | 54.67 | -2.90 | .005 * |
| Target: frontal × MCI | -.02 | .02 | 1564.47 | -.81 | .421 |
| Target: frontoparietal × MCI | -.03 | .02 | 1564.47 | -1.25 | .212 |
| **3-back reaction times** |  |  |  |  |  |
| Intercept | .11 | .06 | 18.78 | 1.95 | .066 |
| Target: frontal | -.05 | .01 | 1580.95 | -3.24 | .001 ** |
| Target: frontoparietal | -.02 | .01 | 1580.34 | -1.67 | .095 |
| Group | -.04 | .06 | 60.57 | -.59 | .561 |
| Gender (M) | -.15 | .06 | 54.62 | -2.51 | .015 * |
| Target: frontal × MCI | .03 | .03 | 1581.02 | 1.31 | .191 |
| Target: frontoparietal × MCI | -.03 | .03 | 1582.21 | -1.11 | .266 |

*Note:* Target: sham is a reference category, Group has two levels: HE (reference in interactions) and MCI. SE = Standard Error, df = Degrees of Freedom, t = t-value, p = p-value. The effects of age and education were regressed out prior to LMM fitting to reduce model complexity and preserve degrees of freedom, given the limited sample size. Gender was incorporated as a covariate into the models. The Conditional and Marginal R^2^ increased in comparison to the un-regressed models in the main results section:

2-back task accuracy: Conditional R^2^ = .46, Marginal R^2^ = .06;

3-back task accuracy: Conditional R^2^ = .44, Marginal R^2^ = .08;

2-back task RT: Conditional R^2^ = .62, Marginal R^2^ = .08;

3-back task RT: Conditional R^2^ = .59, Marginal R^2^ = .06;

1. Bark L, Larsson IM, Wallin E, Simrén J, Zetterberg H, Lipcsey M, et al. Central nervous system biomarkers GFAp and NfL associate with post-acute cognitive impairment and fatigue following critical COVID-19. Sci Rep. 2023 Aug 12;13(1):13144.

2. Pereira JB, Janelidze S, Stomrud E, Palmqvist S, van Westen D, Dage JL, et al. Plasma markers predict changes in amyloid, tau, atrophy and cognition in non-demented subjects. Brain. 2021 Jun 2;144(9):2826–36.

3. Gerards M, Schild AK, Meiberth D, Rostamzadeh A, Vehreschild JJ, Wingen-Heimann S, et al. Alzheimer’s Disease Plasma Biomarkers Distinguish Clinical Diagnostic Groups in Memory Clinic Patients. Dement Geriatr Cogn Disord. 2022;51(2):182–92.

4. Palmqvist S, Janelidze S, Quiroz YT, Zetterberg H, Lopera F, Stomrud E, et al. Discriminative Accuracy of Plasma Phospho-tau217 for Alzheimer Disease vs Other Neurodegenerative Disorders. JAMA - Journal of the American Medical Association. 2020 Aug 25;324(8):772–81.

5. Tissot C, Therriault J, Kunach P, L Benedet A, Pascoal TA, Ashton NJ, et al. Comparing tau status determined via plasma pTau181, pTau231 and [^18^F]MK6240 tau-PET. EBioMedicine. 2022 Feb 1;76:103837.

6. McKeith IG, Ferman TJ, Thomas AJ, Blanc F, Boeve BF, Fujishiro H, et al. Research criteria for the diagnosis of prodromal dementia with Lewy bodies. Neurology. 2020 Apr 28;94(17):743–55.
